# TC14012 enhances the anti-fibrosis effects of UC-MSCs on the liver by reducing collagen accumulation and ameliorating inflammation

**DOI:** 10.1186/s13287-024-03648-w

**Published:** 2024-02-16

**Authors:** Fan Ding, Yuting Liu, Jia Li, Xiao Wei, Jiangdong Zhao, Xiaojing Liu, Liqiang Zhang

**Affiliations:** 1 Institute for Stem Cell and Regenerative Medicine, The Second Affiliated Hospital of Xi’an Jiaotong University, Xi’an, 710004 China; 2 The Key Laboratory of Aerospace Medicine, Ministry of Education, Air Force Medical University, Xi’an, 710032 Shaanxi China; 3 Department of Infectious Disease, The First Affiliated Hospital of Xi’an Jiaotong University, Xi’an, 710061 China

**Keywords:** Liver fibrosis, UC-MSCs, TC14012, LSEC, HSCs, Immunoregulation

## Abstract

**Background:**

Mesenchymal stem cells (MSCs) are attracting attention as a promising cell-based therapy for the treatment of liver fibrosis or cirrhosis. However, the strategies and potential mechanisms of MSCs therapy need further investigation. The CXCL12/CXCR4/CXCR7 chemokine axis is well known to regulate cell migration and is involved in the regulation of liver fibrosis. This study aims to treat MSCs with a CXCR7-specific agonist to evaluate its therapeutic effects on hepatic fibrosis and potential mechanisms.

**Methods:**

TC14012, a potent agonist of CXCR7, has been used to pretreat human umbilical cord-derived MSCs (UC-MSCs) and assess its effect on proliferation, apoptosis, migration, immunoregulation, and gene regulatory network. Then, CCl_4_-induced liver fibrosis mice models were used to evaluate the therapeutic effect and mechanism of TC14012-treated UC-MSCs for treating hepatic fibrosis.

**Results:**

TC14012 increased CXCR7 expression in UC-MSCs. Notably, co-culture of liver sinusoidal endothelial cells (LSEC) with TC14012-pretreated UC-MSCs increased CXCR7 expression in LSEC. Additionally, TC14012 promoted cell migration and mediated the immunoregulation of UC-MSCs. Compared to UC-MSCs without TC14012 pretreatment, UC-MSCs treated with TC14012 ameliorated live fibrosis by restoring CXCR7 expression, reducing collagen fibril accumulation, inhibiting hepatic stellate cells activation, and attenuating the inflammatory response.

**Conclusion:**

This study suggests that TC14012 pretreatment can enhance the therapeutic effects of UC-MSCs on liver fibrosis, mainly by promoting the migration and immunoregulation of MSCs.

**Supplementary Information:**

The online version contains supplementary material available at 10.1186/s13287-024-03648-w.

## Introduction

Liver fibrosis occurs in response to various harmful stimuli leading to excessive deposition of extracellular matrix (ECM) (mainly collagen). Additionally, repeated or persistent injury induces a chronic inflammatory response in the liver by releasing many inflammatory factors, including interleukin (IL)-1β, transforming growth factor (TGF)-β, and tumor necrosis factor (TNF)-α, and disrupting the normal structure of the liver [1, 2]. Several studies revealed a common molecular mechanism underlying liver fibrosis and cirrhosis. Liver sinusoidal endothelial cells (LSEC) have complete differentiation, contain fenestrations, and possess a high degree of endocytic activity. Under normal conditions, LSEC promote the quiescence of hepatic stellate cells (HSCs) by releasing paracrine factors, such as nitric oxide [3]. LSEC dedifferentiation does not prevent or reverse HSCs activation in liver fibrosis. During liver injury, immune cells produce pro-inflammatory cytokines, chemokines, and profibrotic factors, activating HSCs. Activated HSCs migrate to the injury site and secrete ECM, a crucial contributor to fibrosis. Even though several drugs have been developed to ameliorate liver fibrosis based on the etiology, stage, and progression pattern [4, 5], no medications have been approved worldwide for treating liver fibrosis.

Mesenchymal stem cells (MSCs) have recently been identified as a potential therapeutic strategy for liver fibrosis or cirrhosis due to their strong paracrine and immunomodulatory functions. Furthermore, different sources of MSCs may have additional therapeutic potentials, significantly improving therapeutic outcomes according to the pathogenesis of fibrosis. Allogeneic bone marrow mesenchymal stem cells (BMMSCs) have been successfully used in clinical studies to treat cirrhosis [6, 7]. BMMSCs transplantation was utilized in a phase 2 clinical trial on patients with alcoholic cirrhosis. After six months of treatment, the Child–Pugh scores of patients who received BMMSCs were significantly lower than those of the control group [7]. We compared the efficacy of BMMSCs, UC-MSCs, and stem cells from human exfoliated deciduous teeth for treating liver fibrosis. Our unpublished findings showed that UC-MSCs had the best anti-fibrotic effects. Studies have shown that UC-MSCs transplantation is an effective treatment for liver diseases due to UC-MSCs accessibility, easy culture, and low immunogenicity [8, 9]. A study used UC-MSCs to treat hepatitis B virus-induced end-stage liver cirrhosis. The mortality rate in the conventional treatment group was 47.4% after 3 months, while 20.8% in the UC-MSCs transplantation group [8]. Additionally, UC-MSCs efficiently improved liver fibrosis in rodent models [10, 11]. However, the anti-fibrosis still require further investigation.

The cytokines, especially chemokines, play critical roles in liver fibrosis. The chemokine CXCL12, also known as stromal cell-derived factor 1 (SDF-1), has numerous functions in the liver and is significantly upregulated in liver fibrosis [12, 13]. The CXCL12/CXCR4 (a CXCL12 receptor) axis modulates liver fibrosis by promoting the activation and the proliferation of the HSCs [12]. Small molecule inhibitors of CXCR4 have made it a promising target for anti-fibrotic treatment [14, 15]. Recent studies have indicated that inhibiting the CXCL12/CXCR4 axis does not improve liver fibrosis and may even worsen it [16, 17]. These studies suggest that CXCR4 inhibition alone may not restore liver function in fibrosis.

CXCR7 is another important receptor of CXCR12 that counteracts CXCR4 and promotes liver regeneration. In mice with an endothelial cell-specific knockdown of CXCR7, CCl_4_ decreased hepatocyte proliferation and exacerbated liver injury [14]. In particular, injection of TC14102, a specific agonist of CXCR7, downregulated α-SMA and prevented collagen I deposition in wild-type mice but not in CXCR7-specific knockdown mice [14]. Therefore, CXCR7 appears to promote liver regeneration and have anti-fibrotic properties. Many studies have demonstrated that CXCR7 modulates the therapeutic effects of MSCs by promoting their migration [13, 18]. UC-MSCs overexpressing CXCL9 were administrated to evaluate the therapeutic impact on CCl_4_-induced liver fibrosis. CXCL9 overexpression enhanced the homing of UC-MSCs to injury sites of the liver, improving the efficacy of MSCs in treating liver fibrosis [19]. Studies have shown that TC14012 increases CXCR7 expression in cells or tissues [20, 21]. This may improve the therapeutic potential of MSCs for liver fibrosis.

TC14012 is a potent agonist of CXCR7 and an antagonist of CXCR4, which has anti-fibrotic, anti-tumor and pro-vascular regeneration properties [22, 23]. In this study, we used TC14012 as a CXCR7 agonist to promote the anti-fibrotic effects of MSCs. This study proposes a novel approach to facilitate the migration and homing of UC-MSCs in liver fibrosis and other chronic liver ailments.

## Materials and methods

### Animals

Considering that male mice or rats have been predominantly used in previous studies of CCl_4_-induced liver fibrosis models, male C57BL⁄6 mice (aged 6–8 weeks, 18–22 g) were purchased from the Animal Experimental Center of Xi'an Jiaotong University and were offered unrestricted access to food and water under specific pathogen-free conditions at 22–24 °C with 12-h light/dark cycle. All animal procedures and manipulations were conducted under the guidance of the Animal Care Committee of Xi'an Jiaotong University, and the experimental protocols were approved. All animal experiments have been reported in accordance with the ARRIVE guidelines 2.0.

### CCl_4_-induced liver fibrosis and treatment

Group assignments: A total of 24 mice were randomly divided into four groups, including Group 1: WT group (healthy control), Group 2: CCl_4_ with PBS treatment group (treatment control), Group 3: CCl_4_ with UC-MSCs treatment group (CCl_4_ + UC), Group 4: CCl_4_ with UC-MSCs-pretreated with TC14012 group (CCl_4_ + UC-TC), each group containing 6 mice.

#### Experimental design

To induce a liver fibrosis model, mice were administered 5 mL/kg of 20% CCl_4_ (Shanghai Jiahe Biotechnology, Shanghai, CHN) dissolved in olive oil by intraperitoneal injection, twice weekly for 6 weeks. Meanwhile, WT group received the same amount of olive oil. All the mice received similar food and water while residing in the same housing environment. Model mice were randomly divided into different treatment groups: UC-MSCs (CCl_4_ + UC), UC-MSCs pretreated with TC14012 (CCl_4_ + UC-TC), and PBS control (CCl_4_) groups. Considering that tail intravenous injections are less invasive and easier to perform, and that there is no significant difference between portal and tail vein injections for transplantation of MSCs homing to the liver [24, 25], mice received equal amounts of cells (1 × 10^6^ cells suspended in 200 μL PBS) through a single injection in the tail. In the CCl_4_ + UC-TC group, UC-MSCs were pretreated with TC14012 for 24 h to treat liver fibrosis. Mice in the CCl_4_ group were injected with an equal volume of PBS. The mice were anesthetized with 35–45 mg/kg pentobarbital sodium by intraperitoneal injection after 9 days of UC-MSC interventions for sample collection while their circulation and spontaneous breathing were maintained. Blood samples were collected through the inferior vena cava for biochemical analysis. The tissues were collected for histological analysis. Following tissue collection, the mice were euthanized by manual cervical dislocation.

### UC-MSCs culture and treatment

Primary human UC-MSCs were provided by Cyagen Biosciences Co. (Suzhou, CHN). Briefly, the UC-MSCs were cultured with α-MEM medium (Gibco, USA) containing 10% FBS (Sijiqing, CHN), 2 mM L-glutamine (Gibco, USA), 100 U/mL penicillin/streptomycin (Beyotime, CHN), and incubated at 37 °C with 5% CO_2_. UC-MSCs were passaged twice or three times a week. UC-MSCs were stimulated with 5 μM or 30 μM TC14012 (a specific agonist of CXCR7, Sigma, SML3343, USA) for 24 h; then, proteins were extracted for subsequent experiments.

For cell migration, the UC-MSCs were treated with or without 30 μM TC14012 for 24 h, seeded in 0.8-μm pore-size transwell chambers, and cultured for 12 h. To observe, UC-MSCs were stained with 1% crystal violet, and images were taken using a microscope. To assay cell cycle or cell apoptosis, the UC-MSCs were treated with or without 30 μM TC14012 for 24 h, and then cells were harvested and examined by flow cytometry (Beckman CytoFLEX S, USA). Cell cycle or cell apoptosis assays were performed with a Cell Cycle and Apoptosis Analysis Kit (Yeasen, 40301ES60, CHN), or an Annexin V-FITC/PI Apoptosis Detection Kit (Yeasen Biotechnology, 40302ES50, CHN) according to the manufacturer’s instructions.

### LSEC co-cultured with UC-MSCs

2 × 10^5^ UC-MSCs were seeded in 0.4-μm pore-size transwell chambers of a 6-well plate and pretreated with or without TC14012 for 24 h, and then the chambers contained UC-MSCs were inserted into 6-well plates with primary LSEC from fibrotic mice. After co-culture with UC-MSCs for 48 h, Western blotting and immunofluorescent staining were used to measure CXCR7 expression in primary LSEC.

### RNA sequencing and analysis

RNA was isolated from UC-MSCs treatment with or without TC14012 (30 μM) after 24 h by performed with the Direct-zol RNA MiniPrep Kit (Zymo Research). The RNA sequencing libraries were prepared using the mRNAseq Library Prep Kit for Illumina® (NEB England BioLabs). An Illumina Novaseq 6000 was used to sequence fragmented and randomly primed paired-end library pairs. Three biological replicates were sequenced for each group. The analysis of gene expression enrichment, heat maps, and gene set enrichment analysis were generated using clusterProfiler.

### Sirius red staining

After killing mice, their livers were collected, fixed in 4% paraformaldehyde, dehydrated with 30% sucrose, and embedded in OCT for sectioning. Next, 10-μm-thick cross sections were prepared for staining with Sirius Red. Briefly, the sections were washed twice with PBS and incubated with Sirius Red solution (Servicebio, CHN) for 12 min at room temperature. Subsequently, the sections were rinsed twice in absolute ethyl alcohol. Images of Sirius Red were obtained using a microscope and were analyzed quantitatively using Image J software.

### Immunochemical staining

The primary LSEC from fibrotic mice were seeded on confocal dishes. Cells were fixed with 4% paraformaldehyde at 4 °C for 10 min. The livers were fixed with 4% paraformaldehyde at 4 °C for 6 h, dehydrated with 30% sucrose or gradient ethanol, embedded with optimal cutting temperature compound or paraffin, and sectioned into 10-μm-thick cross sections. For immunofluorescence or immunohistochemical assay, the samples were washed with PBS, then incubated with 0.3% triton-100 at 37 °C for 10 min, and incubated with PBS containing 1% BSA at 37 °C for 30 min. In addition, hydrogen peroxide was used to eliminate endogenous peroxidase. Next, the samples were incubated with primary antibodies, CXCR7 (1:200, Proteintech, 20423–1-AP, CHN), Stabilin2 (1:200, MBL, D317-3, JPN), α-SMA (1:200, Abcam, ab5694, UK), IL-1β (1:100, Proteintech, 26048-1-AP, CHN) overnight at 4 °C followed by fluorescent secondary antibodies or horseradish peroxidase (HRP) secondary antibodies to develop color. Images were captured using a laser scanning confocal microscope (Olympus FluoViem FV 1000, Tokyo, JPN) or a phase microscope, and signals were analyzed quantitatively using Image J software.

### Statistical analysis

For all experiments, the sample size was equal to or greater than 3, and the data were presented as the mean ± SD. Statistical analysis was conducted using Student’s *t*-test or one-way ANOVA by Prism software (GraphPad). *P* values less than 0.05 were considered significant.

Other materials and methods please see the additional file 1.

## Results

### The expression of CXCR7 in fibrotic liver and LSEC

**Fig. 1:**
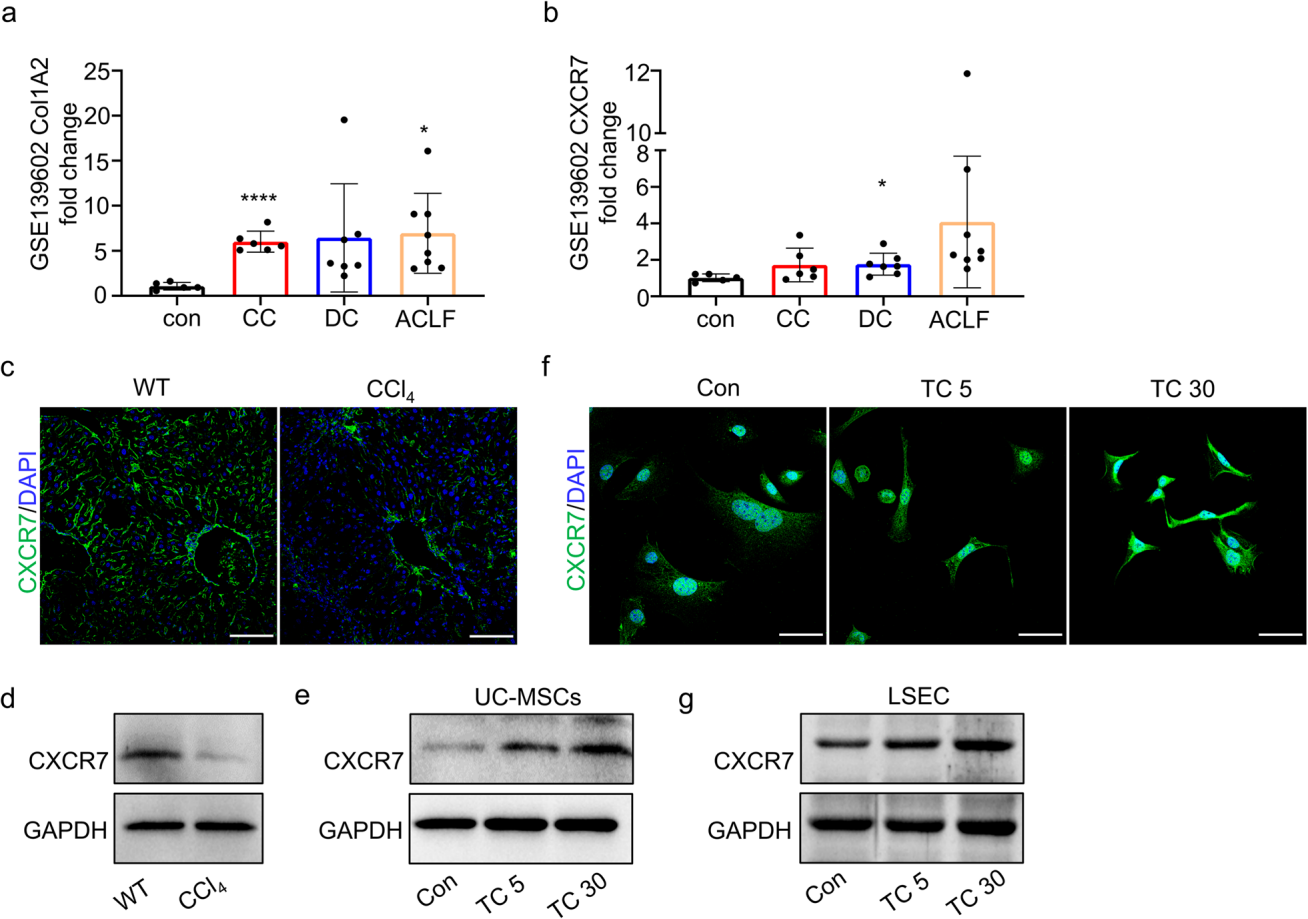
The CXCR7 gene expression profiles in liver and cells. **a** and **b** The Col1A2 gene expression (**a**) and CXCR7 (**b**) in liver biopsy samples from cirrhotic patients by online GEO database. **c** Immunofluorescent staining detected CXCR7 expression in liver. Scale bar: 100 μm. **d** Western blotting detected CXCR7 expression in primary LSEC from normal and liver fibrotic mice. **e** UC-MSCs were treated with TC14012, CXCR7 expression in UC-MSCs were examined by Western blotting. **f** and **g** After LSEC co-cultured with TC14012-pretreated UC-MSCs for 48 h, CXCR7 expression in LSEC were detected by Western blotting and immunofluorescent staining. Scale bar: 50 μm. Full-length blots are presented in Additional file 1: Fig. S8 The data were presented as the mean ± SD. * *p* < 0.05, **** p < 0.0001

Studies have shown that CXCR7 has anti-fibrosis and pro-regeneration potential in chronic liver injury. Excessive synthesis and deposition of collagen-rich ECM (predominantly type 1 collagen, Col1) is an essential hallmark of liver fibrosis and cirrhosis [26, 27]. To investigate the relationship between Col1 and CXCR7 in liver fibrosis and cirrhosis, we analyzed CXCR7 expression in liver biopsy samples from cirrhotic patients using an online database. Additionally, we measured CXCR7 expression in the mice model of CCl_4_-induced liver fibrosis. The analysis of GEO dataset (https://www.ncbi.nlm.nih.gov/geo/query/acc.cgi?acc=GSE139602) showed that the expression of Col1A2 (ID: 11715356_x_at) was significantly increased in patients with liver diseases, including compensated cirrhosis (CC) and acute and chronic liver failure (ACLF) (Fig. 1a and Additional file 1: Fig. S2). This finding was in line with the significantly increased collagen expression in the liver of patients with decompensated cirrhosis (DC) and ACLF, as reported by Graupera et al. [28]. There was an increase in CXCR7 expression in the liver of DC patients compared to healthy controls, with only a weak correlation between CXCR7 and Col1A (Fig. 1b and Additional file 1: Fig. S3). We observed a significant decrease in CXCR7 expression in the liver and LSEC of mice with CCl_4_-induced liver fibrosis compared to the control group (as shown in Fig. 1c, d). These findings are in line with those reported by Ding et al. [14] They also reported that multiple injections of CCl_4_ decreased the abundance of CXCR7-positive cells.

### TC14012 promotes CXCR7 expression in UC-MSCs

No cytotoxicity was observed when treating UC-MSCs with different concentrations of TC14012 (5 to 100 μM), and 30 μM TC14012 increased cell viability (Additional file 1: Fig. S4). Given that TC14012 increases CXCR7 expression in cells or tissues [20, 21], we examined the effect of 5 μM (as low dose) or 30 μM TC14012 on CXCR7 expression in UC-MSCs. Compared to 5 μM TC14012 or control groups, Western blotting indicated that treatment with 30 μM TC14012 significantly increased CXCR7 expression in UC-MSCs (Fig. 1e), suggesting the potential to promote the migration of UC-MSCs.

Since CXCR7 is predominantly expressed in hepatic LSEC, restoring CXCR7 expression improves liver fibrosis and promotes liver regeneration. Hence, LSEC were co-cultured with TC14012-pretreated UC-MSCs in vitro. LSEC co-cultured with 30 μM TC14012-pretreated UC-MSCs notably increased CXCR7 expression in LSEC (Fig. 1f, g). Thus, 30 μM TC14012 were used in subsequent experiments. The above results indicate that TC14012 pretreatment promoted CXCR7 expression in UC-MSCs, especially, UC-MSCs have the potential to regulate CXCR7 expression on LSEC.

### TC14012 enhances migration and immunoregulation of UC-MSCs

**Fig. 2:**
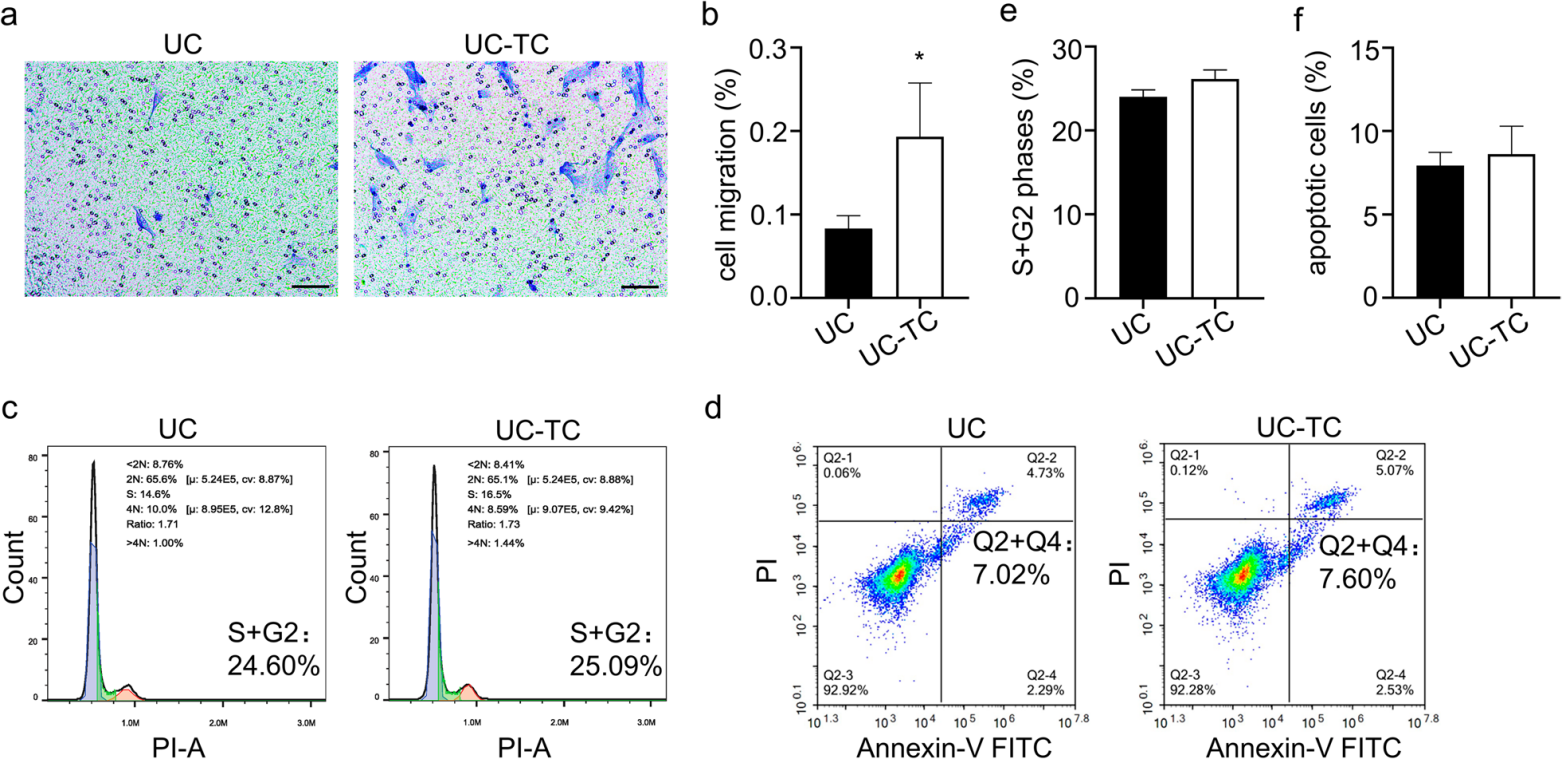
The effects of TC14012 on UC-MSCs bio-behaviors. **a** and **b** Transwell migration and quantitative analysis the effect of TC14012 on UC-MSCs migration. **c** and **e** Analysis the effect of TC14012 on cell cycle of UC-MSCs were performed by flow cytometry. **d** and **f** Analysis the effect of TC14012 on cell apoptosis of UC-MSCs were performed by flow cytometry. The data were presented as the mean ± SD. * p < 0.05

Studies have shown that upregulation of CXCR7 expression facilitates cell migration [18, 29]. The result of transwell migration experiment showed that TC14012 significantly enhanced UC-MSCs migration compare to the control group (Fig. 2a, b). However, flow cytometry results showed that TC14012 stimulation showed no noticeable effect on cell proliferation and apoptosis of UC-MSCs (Fig. 2c–f).

**Fig. 3:**
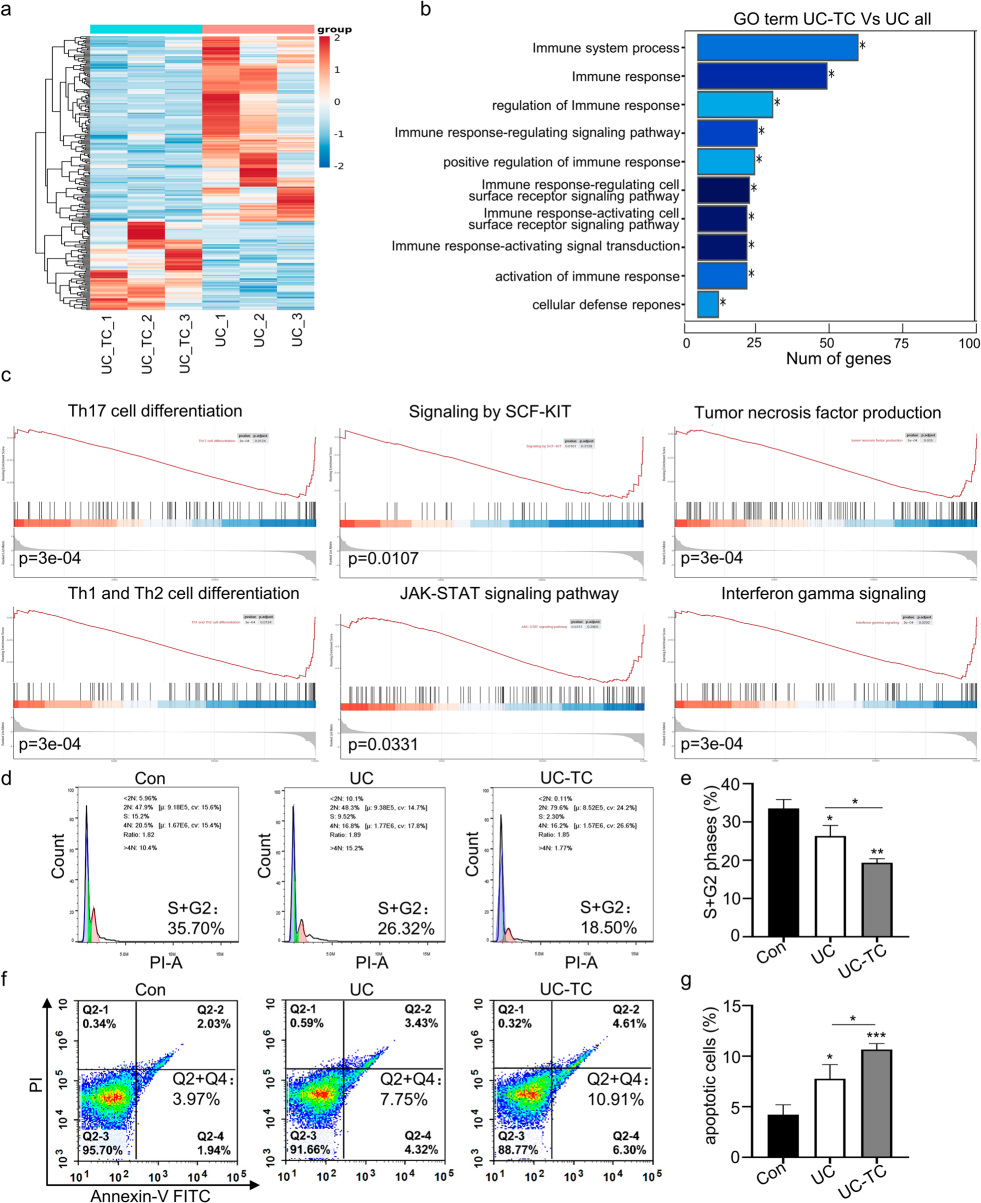
RNA sequencing analyze the effects of TC14012 on gene expression profiles in UC-MSCs. **a** RNA sequencing analysis of the differential genes between UC-MSCs treated with or without TC14012. **b** GO enrichment analysis of the main functions of the difference genes in cell processes. **c** GSEA analyzed the differential genes mainly involved in cell processes and signaling pathways. **d** and** e** The cell cycle and statistical analysis of UC-MSCs induced T lymphocyte proliferation were performed by flow cytometry. **f** and** g** The cell apoptosis and statistical analysis UC-MSCs induced T lymphocyte apoptosis were performed by flow cytometry. The data were presented as the mean ± SD. * *p* < 0.05, ** *p* < 0.01, *** *p* < 0.001

RNA sequencing was performed to evaluate the effect of TC14012 on the gene regulatory network of UC-MSCs. RNA sequencing showed that 75 genes were upregulated and 158 genes were downregulated in UC-MSCs after exposure to TC14012 (Fig. 3a). Gene ontology (GO) enrichment analysis revealed that these differential genes are mainly involved in the immune response and related signaling pathways (Fig. 3b). Gene set enrichment analysis (GSEA) revealed that differential genes are involved in the inflammatory response, specifically in the regulation of Th1/Th17 differentiation, JAK-STAT and SCF-KIT inflammation-related signaling pathways, and production of pro-inflammatory cytokines TNF-α and INF-γ (Fig. 3c). Meanwhile, genes associated with pro-inflammatory response in UC-MSCs were downregulated by TC14012 treatment (Fig. 3c), suggesting that TC14012 potentially enhances the immunosuppressive function of UC-MSCs. To confirm the immunomodulatory effect of TC14012 on UC-MSCs, a single sample GSEA was performed on genes associated with the immune response. The result showed that these genes are mainly enriched in neutrophils and lymphocytes (Additional file 1: Fig. S5). Some studies suggest that neutrophils may not be directly associated with liver fibrosis [30, 31]. The effects of UC-MSCs on cell cycle and apoptosis of T lymphocytes were determined by co-culturing activated T lymphocytes with UC-MSCs. Flow cytometry demonstrated that pretreatment with TC14012 significantly increased the immunomodulatory effects of UC-MSCs, suppressing T lymphocyte proliferation and promoting T lymphocyte apoptosis (Fig. 3d–g). These results suggest that pretreatment with TC14012 improves cell migration and immunoregulation of UC-MSCs.

### TC14012 enhances the anti-fibrotic effects of UC-MSCs

**Fig. 4:**
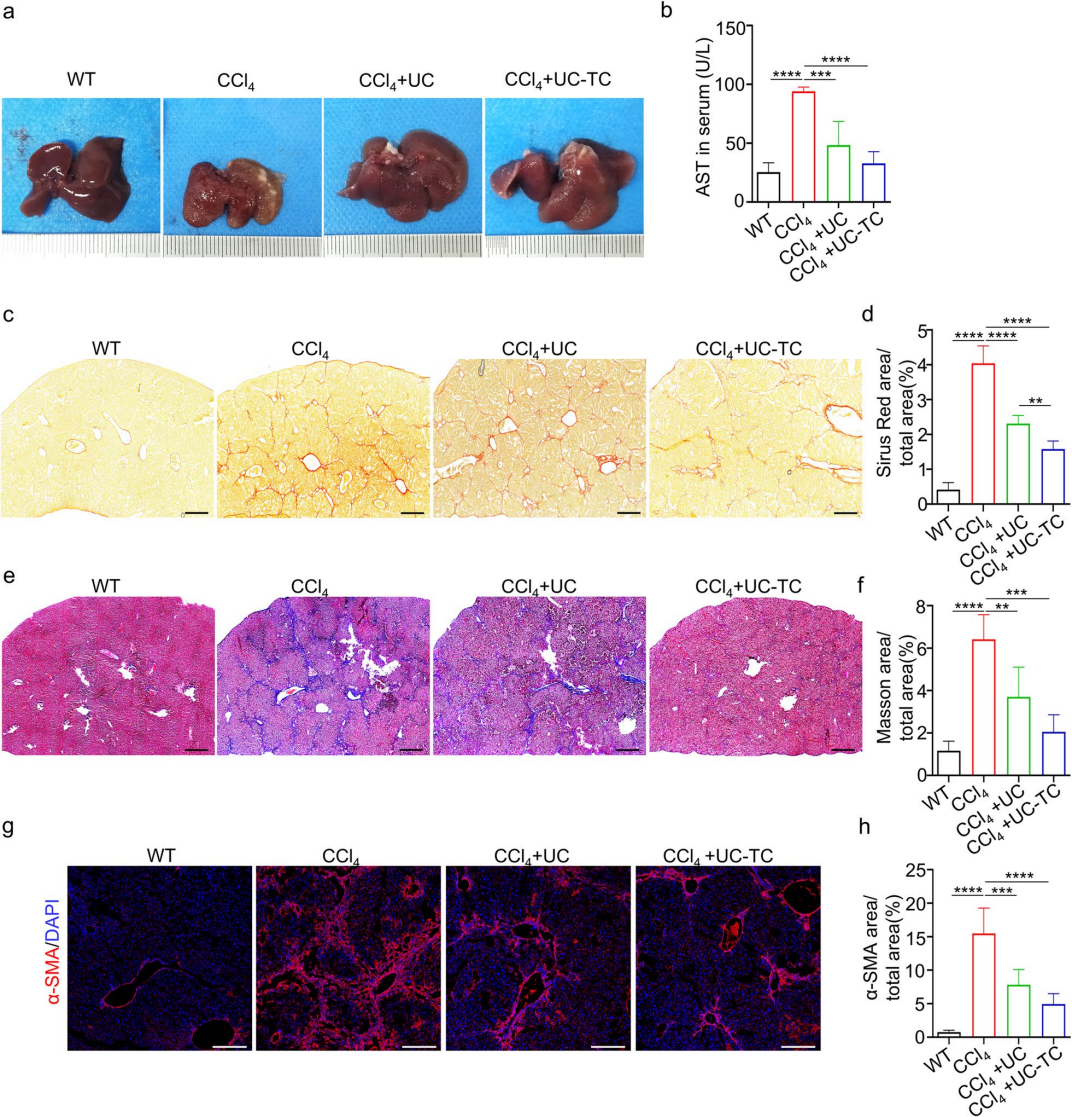
TC14012 enhances the anti-fibrotic effect of UC-MSCs. The CCl_4_-induced liver fibrotic mice were treated with UC-MSCs with or without TC14012 pretreatment. **a** The morphological liver was observed. **b** Liver function was evaluated by examined the serum levels of AST, each group *n* = 6 mice. **c** and **e** The accumulation of collagen fibers in liver were performed by Sirius Red staining (**c**) and Masson trichrome staining (**e**). Scale bar: 500 μm. **g** The activation of HSCs were analyzed in liver by immunofluorescent staining of α-SMA. Scale bar: 200 μm. **d**, **f**, **h** The quantitative analysis of Sirius Red staining (**d**), Masson trichrome staining (**f**), and α-SMA staining (**h**), each group *n* = 6 mice. The data were presented as the mean ± SD. One-way ANOVA and Student’s *t*-test: **p < 0.01, ***p < 0.001, ****p < 0.0001

To evaluate whether TC14012 enhances the anti-fibrotic efficacy of UC-MSCs, CCl_4_-treated mice received UC-MSCs therapy. The morphological measurements showed a smoother liver surface in both UC-MSCs (UC) and TC14012-pretreated UC-MSCs (UC-TC) groups compared to the CCl_4_ control group (Fig. 4a). There was no significant change in liver weight ratio (Additional file 1: Fig. S6a). AST significantly decreased in UC-MSCs-treated mice, particularly in the UC-TC group. However, neither UC nor UC-TC significantly decreased the serum levels of ALT (Additional file 1: Fig. S6b). Sirius Red staining and Masson trichrome staining revealed that UC-TC group had reduced CCl_4_-induced hepatic pseudobullet formation and collagen fiber accumulation compared to the UC-MSCs group (Fig. 4c–f). Similarly, α-SMA staining revealed that HSCs activation decreased (Fig. 4g, h).

**Fig. 5:**
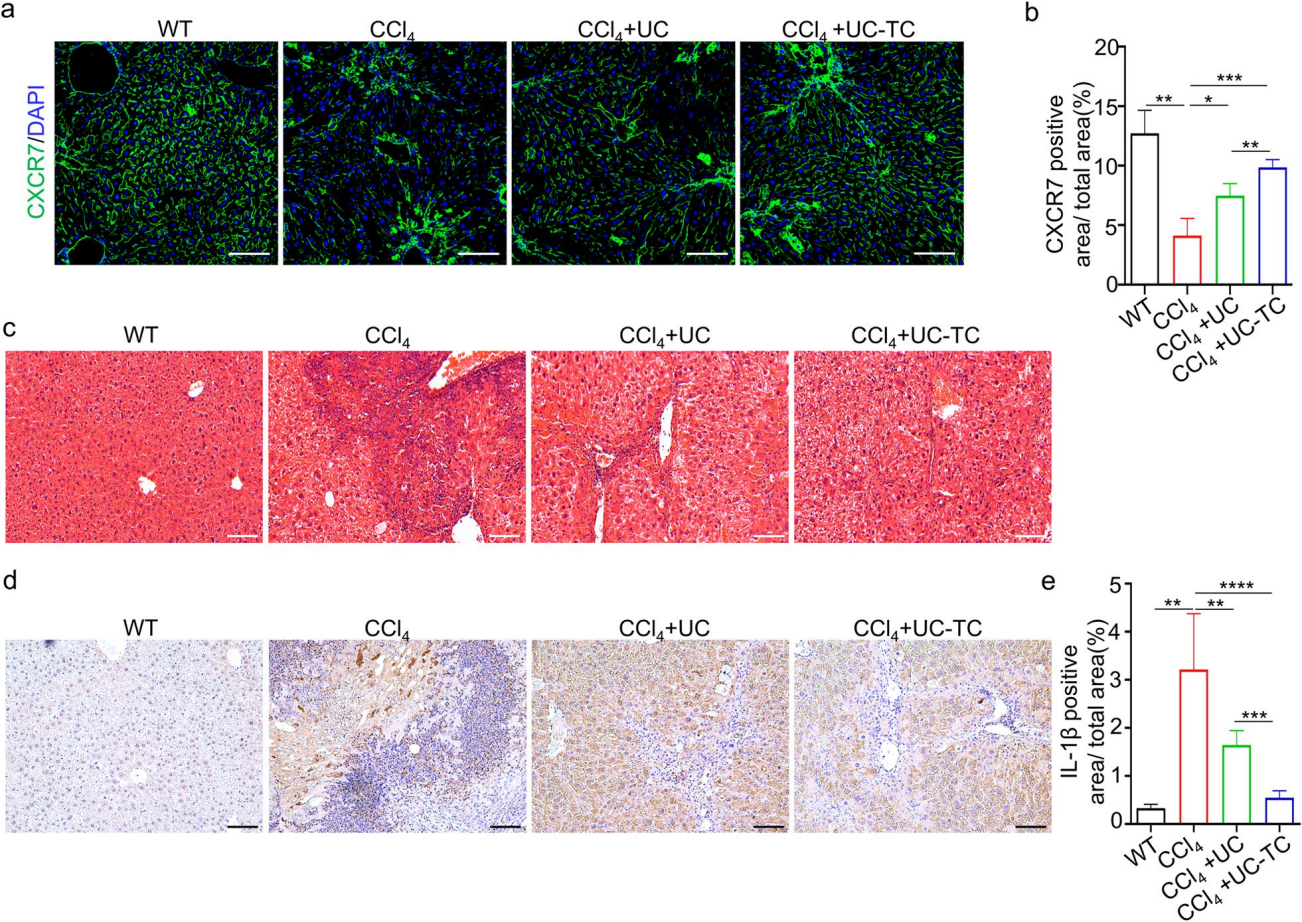
TC14012 contributes UC-MSCs to restoring CXCR7 expression and suppressing inflammation in the mice model of CCl_4_-induced acute liver injury. The CCl_4_-induced liver fibrotic mice were treated with UC-MSCs with or without TC14012 pretreatment. **a** and **b** The CXCR7 expression and quantitative analysis in liver were performed by immunofluorescent staining, each group n = 6 mice. Scale bar: 100 μm. **c** and **d** The inflammatory response in liver were observed by H&E staining (**c**) and immunofluorescent staining of IL-1β (**d**). Scale bar: 100 μm. **e** The quantitative analysis of immunofluorescent staining of IL-1β in liver, each group *n* = 6 mice. The data were presented as the mean ± SD. One-way ANOVA and Student’s t-test: *p < 0.05, **p < 0.01, ***p < 0.001, ****p < 0.0001

Subsequently, we examined the effect of TC14012 pretreated UC-MSCs on CXCR7 expression in fibrotic liver. Our results showed that CXCR7-positive signals were mainly restricted to LSEC, and treatment with UC-MSCs restored the number of CXCR7-positive LSEC, especially in the UC-TC group (Fig. 5a, b, Additional file 1: Fig. S7). This finding suggests that restoration of LSEC and their CXCR7 expression is essential to inhibit HSCs activation and release the fibrotic phenotype. Considering that MSCs are essential for immunoregulation and that TC14012 pretreatment is involved in the immunoregulation of UC-MSCs, the effect of UC-MSCs was examined on liver inflammation. The results of H&E staining showed significant infiltration of inflammatory cells in the CCl_4_ group (Fig. 5c). Notably, the inflammatory microenvironment was significantly improved in TC14012-pretreated UC-MSCs compared to the UC-MSCs group (Fig. 5c). Immunohistochemical staining showed reduced production of the proinflammatory factor IL-1β in the liver (Fig. 5d, e). The results showed that TC14012 improved the anti-fibrotic effects of UC-MSCs by reducing collagen fiber accumulation, restoring CXCR7 expression, and inhibiting inflammatory infiltration.

**Fig. 6:**
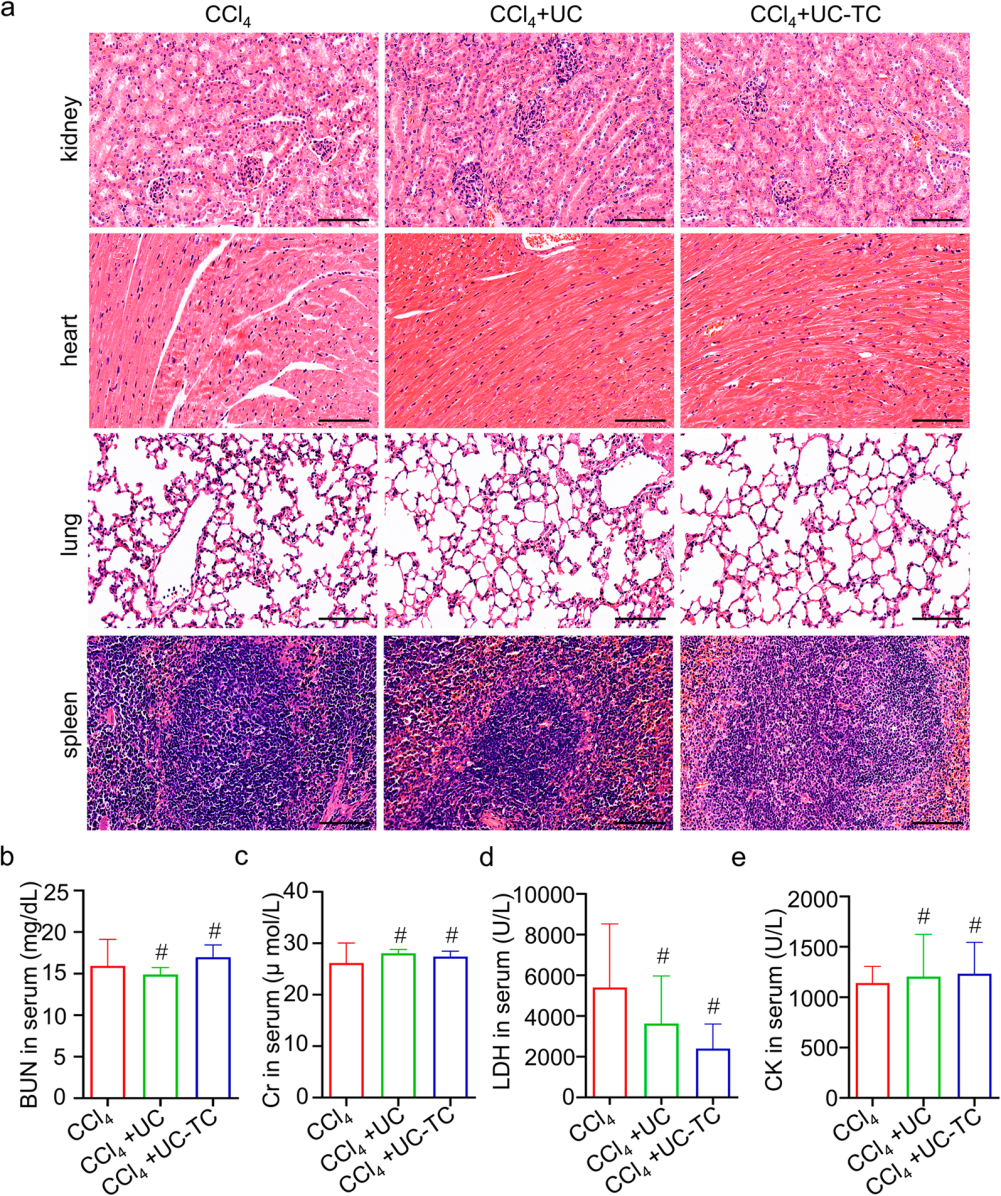
The biosafety assessment of UC-MSCs therapy. **a** The morphological measurements of major organs, including kidneys, hearts, lungs, and spleens in liver fibrotic mice with UC-MSCs therapy were evaluated by H&E staining. Scale bar: 100 μm. **b** and **c** Kidney function was evaluated by examined the serum levels of BUN (**b**) and Cr (**c**). **d** and **e** Myocardial enzyme assays were performed by examined the serum levels of LDH (**d**) and CK (**e**). The data were presented as the mean ± SD. One-way ANOVA: ^#^*p* > 0.05

Finally, the safety of TC14012-pretreated UC-MSCs was evaluated after transplantation. Particularly, we measured morphological and functional effects on major organs. As expected, no immune response and tissue damage were found in the hearts, spleens, lungs, and kidneys in H&E staining (Fig. 6a) and blood cell counts (Additional file 1: Table S1). Moreover, biochemical assays of BUN, Cr, CK, and LDH showed that TC14012-pretreated UC-MSCs are at least as safe as UC-MSCs (Fig. 6b–e).

## Discussion

CXCR7 is a receptor that binds to CXCL12, mainly on endothelial cells (ECs). It has a greater affinity than CXCR4 to CXCL12 [32]. In the mice model of CCl_4_-induced acute liver injury, Ding et al. [14] found that CXCR7 expression was upregulated in LSEC after 2 days of a single injection of CCl_4_. To determine if CXCR7 activation in LSEC contributes to liver regeneration, CXCR7 knockout mice (CXCR7^iΔEC/iΔEC^) were generated by crossing tamoxifen-induced EC-specific Cre ERT2 mice with LoxP site-flanked CXCR7 mice. The results unveiled that compared to controls, hepatocyte proliferation in CXCR7^iΔEC/iΔEC^ mice was significantly impaired after CCl_4_ injury [14], suggesting that CXCR7 activation is essential for liver regeneration.

In chronic liver fibrosis and LSEC repeatedly exposed to CCl_4_, CXCR7 expression is significantly reduced [14], while CXCR4 and CXCL12 expression are upregulated [33]. Notably, injection of a CXCR7-specific agonist TC14012 ameliorated liver fibrosis by reducing the expression of α-SMA and collagen I [33]. Similar effects were also observed in pulmonary fibrosis [22]. By analyzing the GEO dataset (GSE139602), we found that CXCR7 increased in patients with decompensated cirrhosis (DC). Increased CXCR7 expression has been found in human and murine hepatocellular carcinoma (HCC) and promoted the angiogenesis, proliferation, invasion, and migration of HCC cells [34–36]. In specific individuals with advanced cirrhosis, elevated CXCR7 levels may significantly increase the likelihood of developing HCC.

MSCs are somatic stem cells with the characteristics of self-renewal, multilineage differentiation, and immunomodulation. Numerous studies have demonstrated that MSCs therapies have beneficial effects in treating several diseases, including chronic liver disease [37–39]. MSCs secrete a wide range of bioactive substances, including chemokines, cytokines, and growth factors, to enhance growth and homing capacity and attenuate inflammation. Studies indicate that transplanted MSCs improve diseases through paracrine effects, with or without extracellular vesicles, rather than through differentiation capacity [40, 41]. Due to the short half-life of transplanted MSCs in the host, MSCs differentiation is not enough to treat diseases. Therefore, MSCs migration and homing to the injury sites can enhance their beneficial effects.

In preclinical and clinical studies, MSC transplantation mitigated liver fibrosis by restoring liver function and improving liver regeneration [42–44]. In vivo and in vitro experiments showed that MSCs can inhibit HSCs activation by modulating several signaling pathways. For instance, they downregulate TGF-β/Smad, Wnt/β-catenin, and PI3K/Akt pathways and upregulate Notch pathway [45–47]. In addition, MSCs upregulate MMPs (e.g., MMP9 and MMP13) and inhibit the expression of TIMPs (e.g., TIMP-1), which directly degrade ECM to alleviate liver fibrosis [48]. Notably, the homing efficiency of transplanted MSCs to injury sites pivotal to their regenerative and anti-fibrotic effects. Pretreatment of MSCs with various factor can modulate their homing capacity. Studies showed that CXCL9 overexpression by UC-MSCs improves their homing to the injury sites and alleviates liver fibrosis [19]. Notably, CXCR7, a specific receptor of CXCL12, is involved in MSC migration. Shao et al. found that CXCR7 overexpression significantly facilitates MSCs homing to injured sites and attenuates acute lung injury [18].

Immune dysregulation is considered a significant cause of fibrosis and liver injury. MSCs can produce and secrete a large number of cytokines, including IL-6, IL-10, TGF-β, HGF, PGE2, NO, etc. They negatively regulate the function of immune cells, including T lymphocytes, macrophages and neutrophils, and promote the proliferation of anti-inflammatory cells, such as M2 macrophages and Tregs [42, 49, 50]. Several studies found that MSC therapy can ameliorate liver fibrosis and acute liver injury in mice by suppressing the inflammatory response, activating M2 macrophages, inhibiting M1 macrophages, and promoting Tregs expansion [51–53]. Our results showed that TC14012 significantly enhanced the immunomodulatory capacity of UC-MSCs, thereby enabling UC-MSCs to more effectively suppress inflammation, inflammatory infiltration, and IL-1β expression. Moreover, it has also been demonstrated that the inhibitory effects of MSCs on the cell proliferation of CD4^+^ or CD8^+^ T cells were significantly enhanced after serum pretreatment or Foxp3 overexpression [54, 55]. These observations suggest that the immunomodulatory function of MSCs plays a crucial role in alleviating liver fibrosis.

Despite our showing that TC14012 improved the anti-fibrotic effects of UC-MSCs, some issues still need to be addressed. For example, in vitro experiments revealed that TC14012 promoted CXCR7 expression and facilitated of UC-MSCs migration, but whether it promoted UC-MSC homing to the injury sites remains to be confirmed. In addition, the effect of pretreated UC-MSCs on liver regeneration and its intrinsic mechanisms need further investigation. Addressing these above issues can provide potential strategies for the clinical application of MSCs in treating chronic liver diseases, such as liver fibrosis and cirrhosis.

## Conclusion

**Fig. 7:**
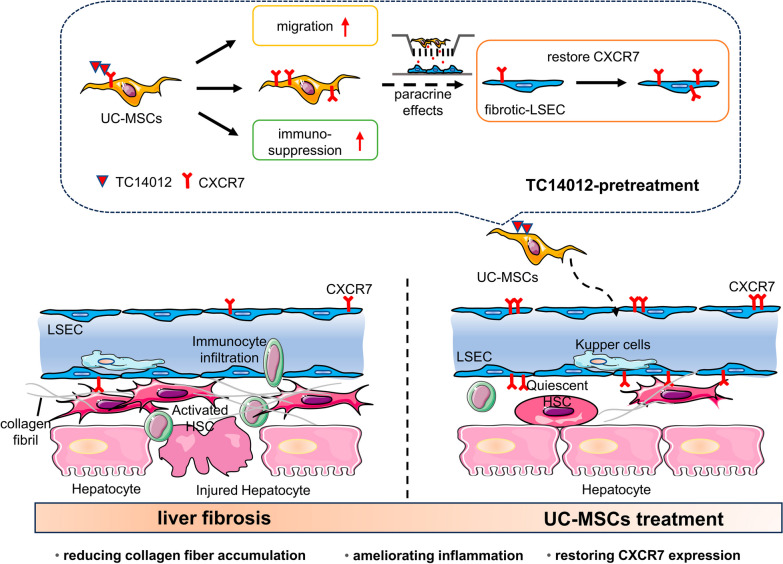
Schematic diagram showing how TC14012 enhances the therapeutic potential of UC-MSCs in liver fibrosis. TC14012 treatment promotes the migration and immunosuppressive function of UC-MSCs. In addition, the increased CXCR7 expression in TC14012 pretreated UC-MSCs could regulate CXCR7 expression in LSEC through paracrine functions. TC14012-pretreated UC-MSCs show better anti-fibrotic effects than UC-MSCs. Various pathological factors stimulate HSCs activation and inflammatory cell infiltration leading to excessive extracellular matrix deposition (especially collagen fibril) and liver damage ultimately contributing to liver fibrosis. TC14012-pretreated UC-MSCs exhibit better anti-fibrotic efficacy compared to UC-MSCs by reducing HSCs activation, alleviating inflammation, and restoring CXCR7 expression

Our study showed that TC14012 significantly promoted the migration of MSCs and extensively regulated genes associated with immune regulation to enhance the immunoregulatory effects of MSCs. In vivo experiments demonstrated that TC14012 enhanced the therapeutic potential of UC-MSCs for liver fibrosis by reducing collagen fiber accumulation, restoring CXCR7 expression, and inhibiting inflammatory infiltration (Fig. 7). Taken together, our results suggest that pretreatment with TC14012 enhances the anti-fibrotic efficacy of MSCs by promoting their migration and immunosuppression. Furthermore, our findings suggest that pretreatment of MSCs with peptides or small molecule drugs can improve their anti-fibrotic efficacy and optimize MSCs therapy. Further studies are needed to investigate how TC14012 regulates MSCs migration and immunomodulation and how TC14012-pretreated MSCs promote CXCR7 expression in LSEC.

### Supplementary Information

**Additional file 1**. Figures and methods and tables.

## Data Availability

We have submitted the raw of RNA sequencing data to the Sequence Read Archive (SRA) of NCBI (accession number PRJNA1002638), which is publicly available at https://www.ncbi.nlm.nih.gov/sra/PRJNA1002638.

## Abbreviation

MSCs: Mesenchymal stem cells
UC-MSCs: Umbilical cord-derived MSCs
BMMSCs: Bone marrow mesenchymal stem cells
LSEC: Liver sinusoidal endothelial cells
HSCs: Hepatic stellate cells
ECM: Extracellular matrix
