# Supplementary material for: TC14012 enhances the anti-fibrosis effects of UC-MSCs on the liver by reducing collagen accumulation and ameliorating inflammation
Source: Stem Cell Res Ther. 2024 Feb 16;15:44. doi: 10.1186/s13287-024-03648-w (PMC10870604; doi:10.1186/s13287-024-03648-w)
Supplement: Supplementary file 1 — Additional file 1. Figures and methods and tables. [file 13287_2024_3648_MOESM1_ESM.docx]

**Supplementary data**

**The characteristics of UC-MSCs and LSEC**

To characterize UC-MSCs, UC-MSCs immunophenotype, clone formation, and osteogenic differentiation capacity were performed. UC-MSCs were incubated with PE- or FITC-conjugated antibodies against human CD31, CD45, CD73, CD90, and CD105, which were examined by flow cytometry. The clone formation units of UC-MSCs were stained with 1% crystal violet, and images were captured using a microscope. UC-MSCs were induced with osteogenic medium for 21 days and were stained with 1% Alizarin red, and images were obtained using a microscope. For characterization of LSEC, LSEC were harvested, fixed in 4% glutaraldehyde, and then dehydrated in a gradient ethanol. Next, the samples were dried, coated with gold, and taken images using a SEM (S-4800, Hitachi).


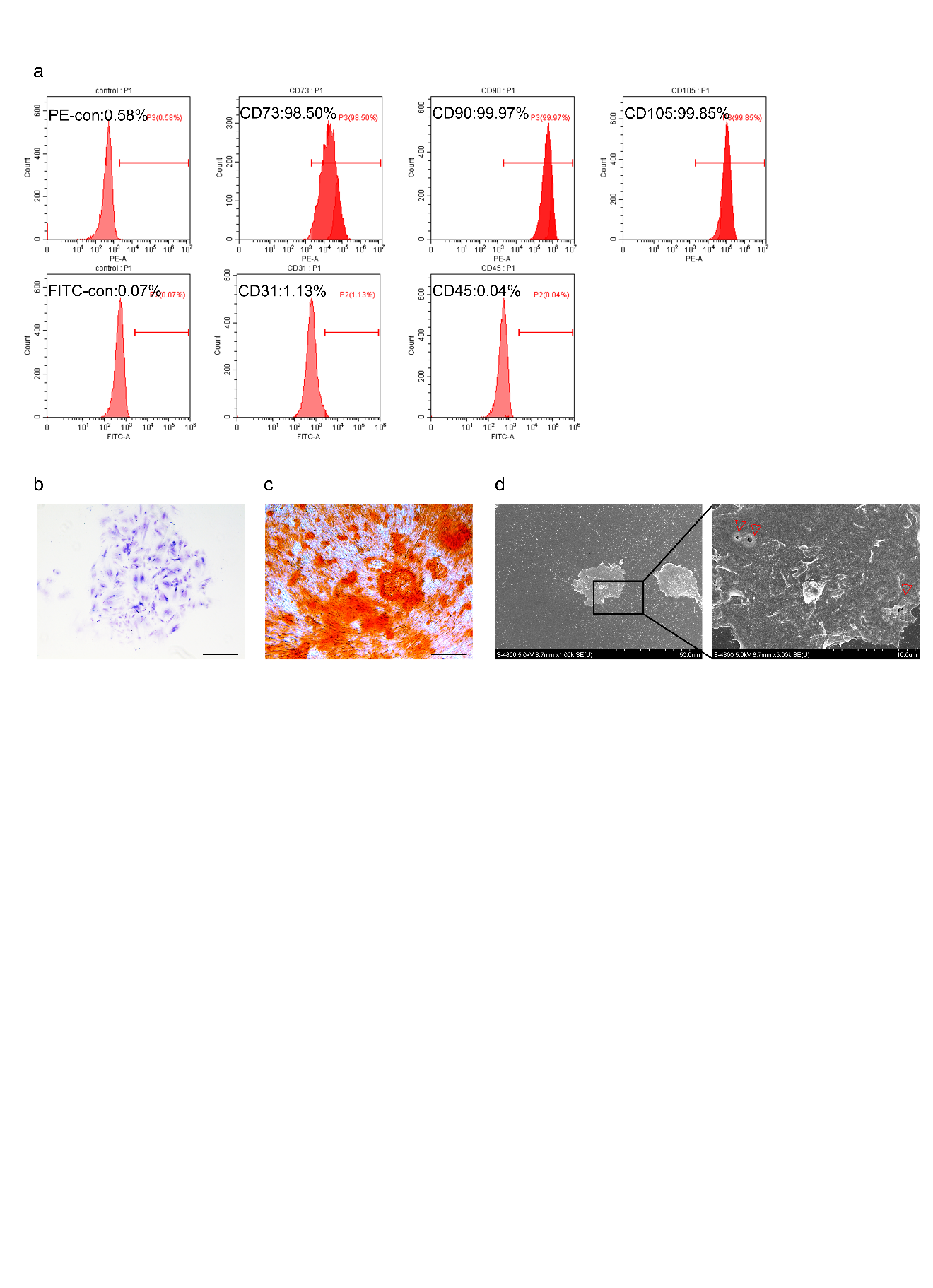


**sFigure 1** The characterization of UC-MSCs and LSEC

**a** The immunophenotype of UC-MSCs was performed by flow cytometry. **b** The clone formation units of UC-MSCs were detected by crystal violet staining. Scale bar: 200 μm. **c** The osteogenic differentiation of UC-MSCs was performed by Alizarin red staining. Scale bar: 200 μm. **d** The fenestra of LSEC were performed by SEM. The triangular symbols displayed the fenestrations on the cell surface of LSEC.

**The expression value of Col1A2 (ID:11715356_x_at) in GSE139602**

Collagen fiber accumulation is an essential hallmark of chronic liver diseases such as liver fibrosis and cirrhosis. To investigate collagen I expression at different stages of liver diseases, we analyzed Col12A expression in liver biopsy samples from patients with compensated cirrhosis, decompensated cirrhosis, and acute chronic liver failure cirrhotic patients using an online database (GSE139602).


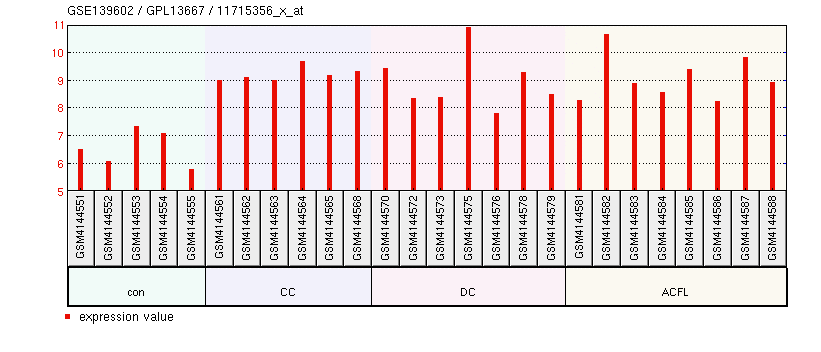
 **sFigure 2** The expression value of Col1A2 in GSE139602 dataset

con: liver biopsy from healthy person; CC: liver biopsy from patients with compensated cirrhosis; DC: liver biopsy from patients with decompensated cirrhosis; ACFL: liver biopsy from patients with acute chronic liver failure.

**Correlation analysis of Col1A2 with CXCR7**

Increased collagen (Type 1 of collagen, Col1) expression is an important hallmark of fibrosis. To investigate the relationship between Col1 and CXCR7 in liver fibrosis and cirrhosis, Col1A2 and CXCR7 expression were analyzed in liver biopsy samples from cirrhotic patients using an online database (GSE139602).


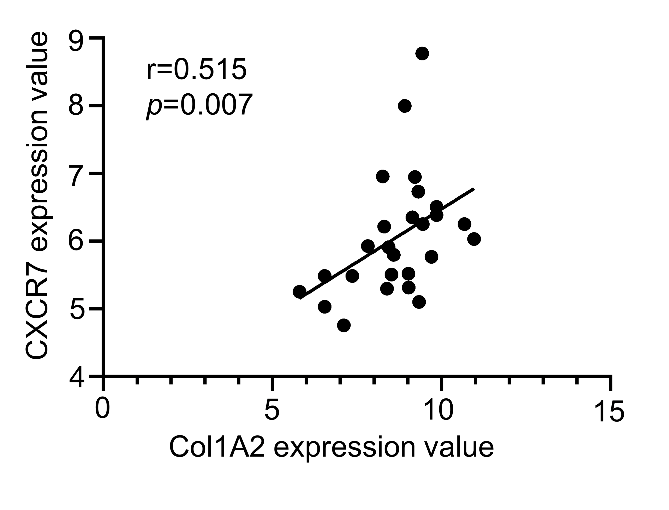


**sFigure 3** Correlation analysis of Col1A2 with CXCR7

A correlation analysis between Col1A2 and CXCR7 was performed by using the expression value of Col1A2 and CXCR7 in the liver obtained from healthy and patients with CC, DC, and ACLF using an online database (GSE139602).

**Screening the concentration of TC14012-pretreated** **UC-MSCs**

UC-MSCs were seeded with 3000 cells in 96-well plates and incubated in an incubator for 12 hours. The next day, the culture medium was added with different doses of TC14012 for 24 h. CCK8 was added and incubated for another 4 hours. The OD value was measured at 450 nm.


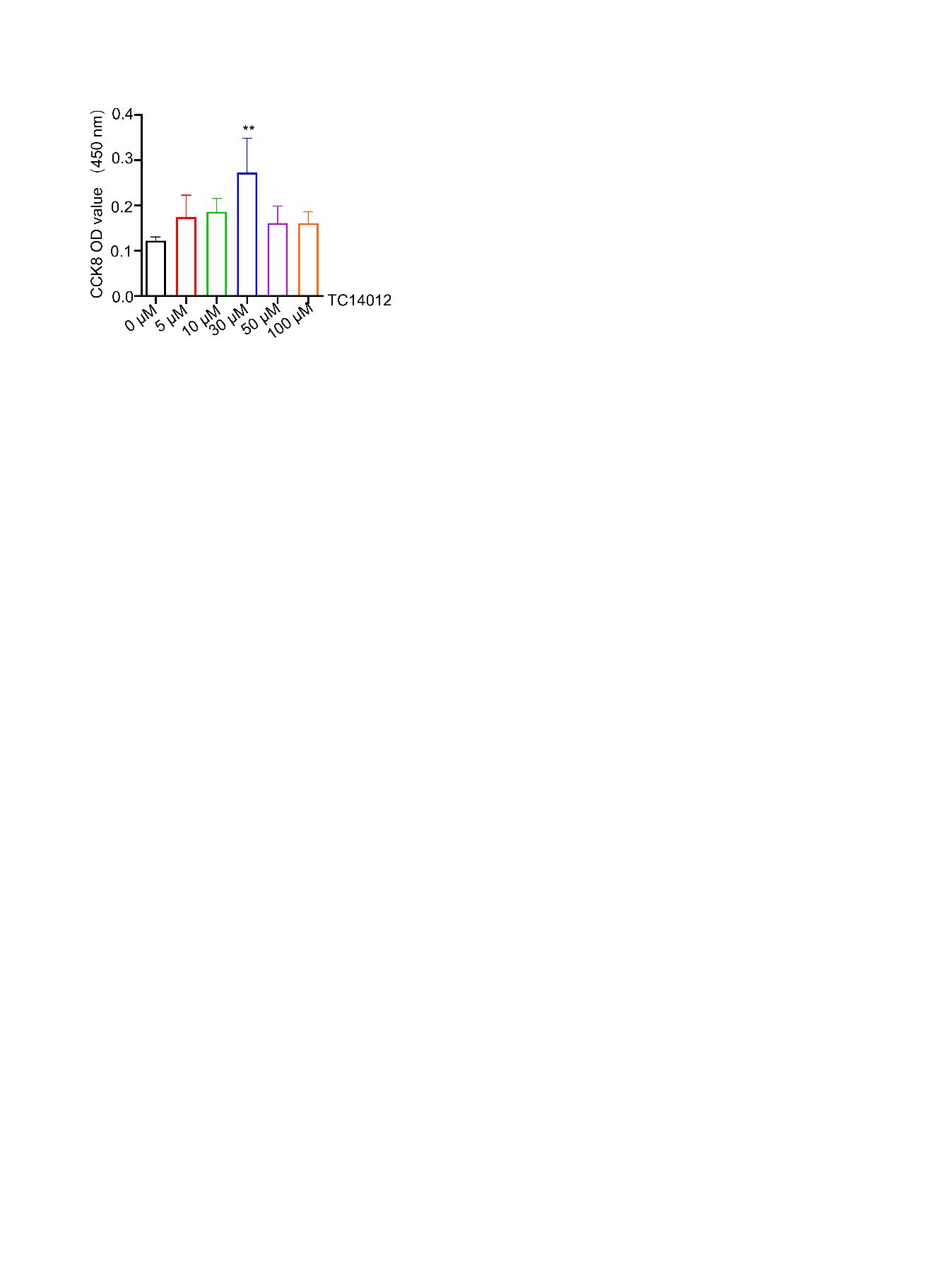


**sFigure 4** The cell viability was tested in UC-MSCs treated with different doses of TC14012 by CCK8 assay. The data were presented as the mean ± SD. ** p < 0.01.

**The gene set enrichment analysis (GSEA) of immune response**

To assess the immunomodulatory effects of TC14012 on UC-MSC, a single sample GSEA was performed on genes associated with the immune response.


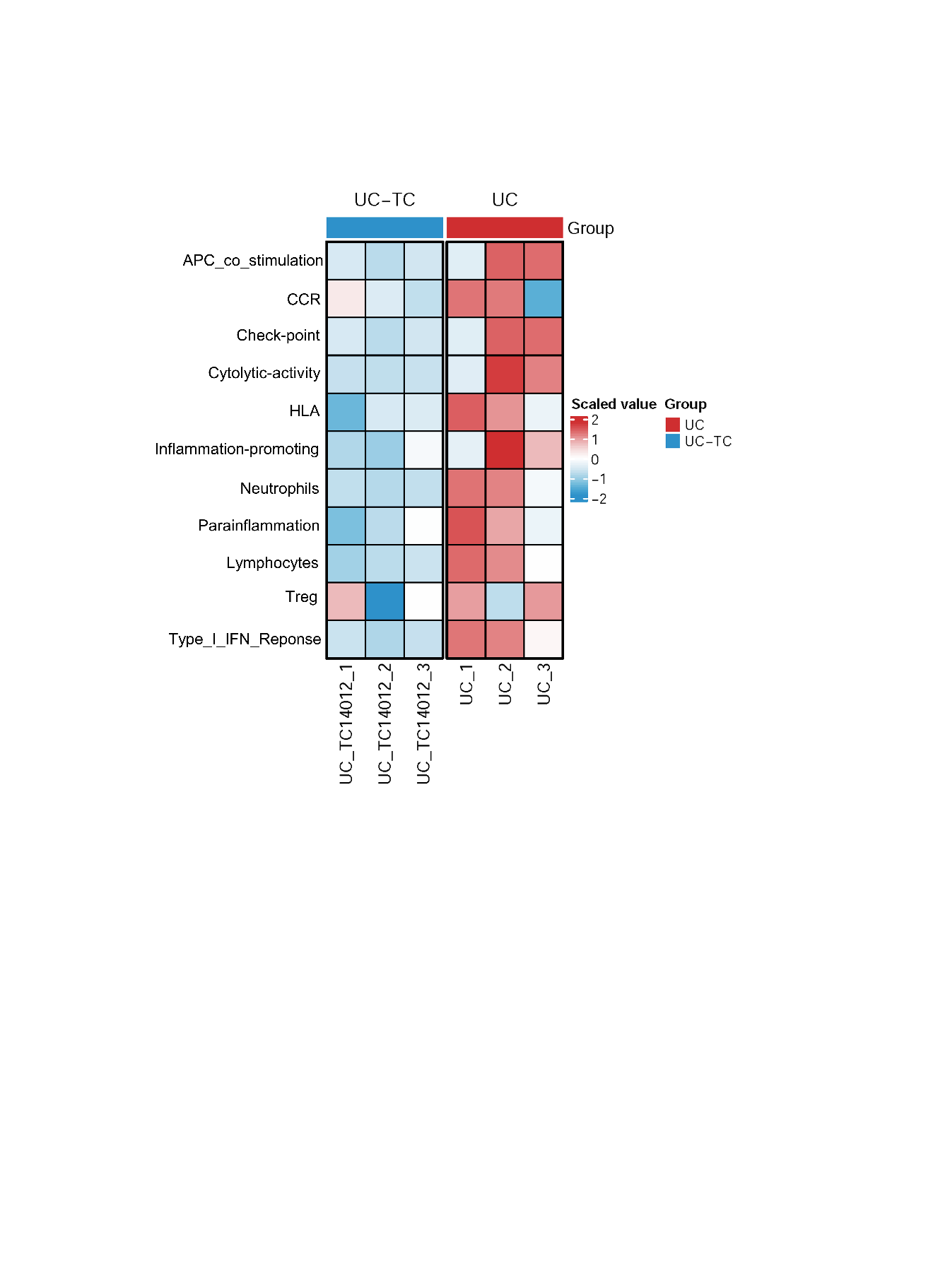


**sFigure 5** GSEA of immune response

GSEA results for immune check-point, HLA, inflammation, and immunocytes including neutrophils, lymphocytes, and Treg cells.

**Liver weight ratio and ALT tests**

Mouse weight and whole liver weight were measured when sacrificed. The ratio of liver weight to body weight was analyzed. For ALT assays, blood was collected immediately at the time of sacrifice and the serum was separated by centrifugation at 5000 g at 4 °C for 10 minutes. Alanine aminotransferase (ALT) was assessed by using the ALT Assay Kit from NJJCBIO Company (NJJCBIO, CHN) according to the manufacturer's instructions.


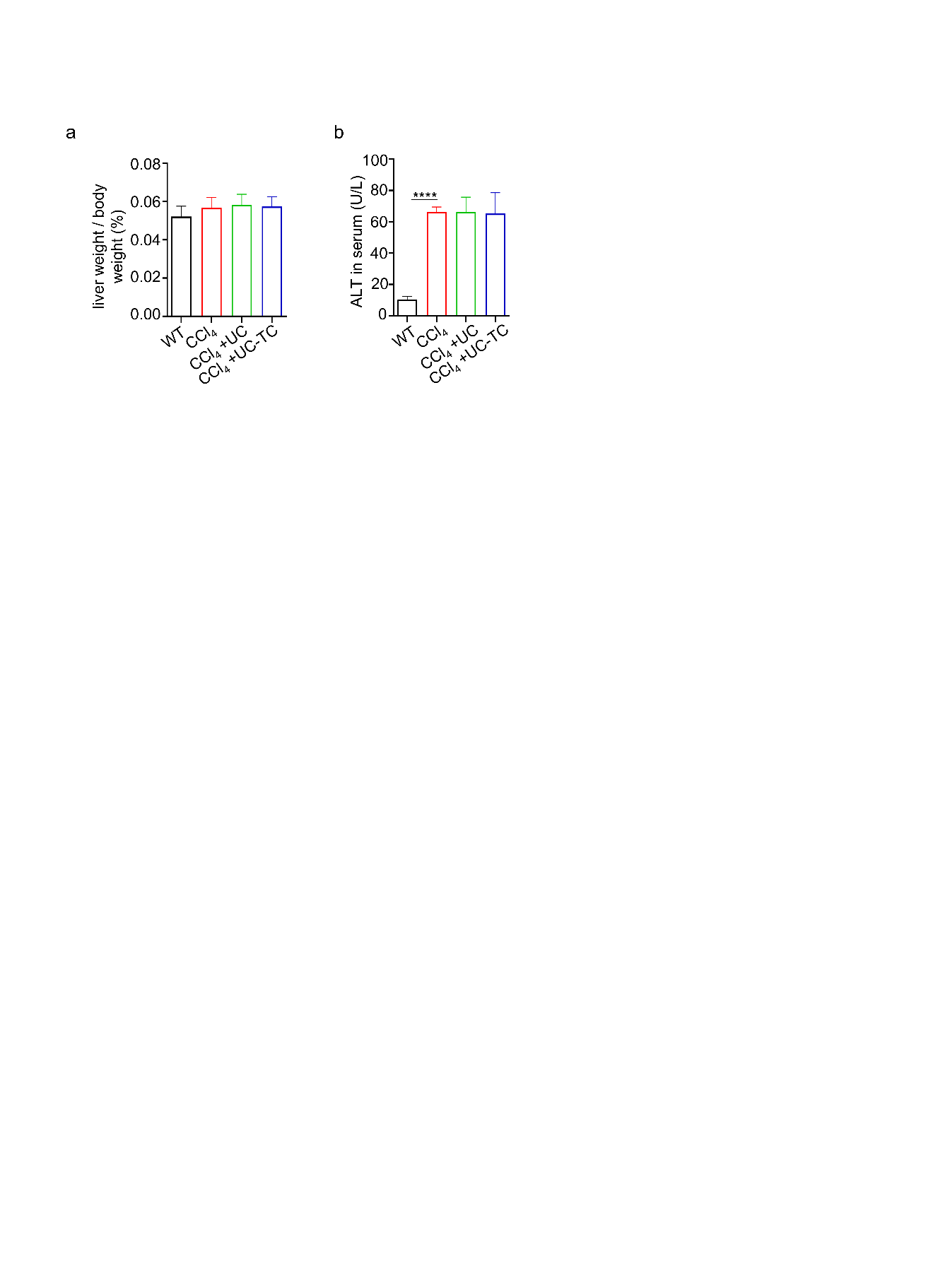


**sFigure 6** Liver weight and serum ALT tests.

**a** The ratio of liver weight to total body weight was evaluated in fibrotic mice treated with UC-MSCs. **b** The liver function was evaluated by examined the serum levels of ALT. The data were presented as the mean ± SD. **** p < 0.0001.

**CXCR7 co-stained with Stabilin2 in liver**

CXCR7 is mainly expressed in endothelial cells or LSEC in the liver. To evaluate whether UC-MSCs treatment increased CXCR7 expression in LSEC, CXCR7 was co-stained with Stabilin2, a specific biomarker of LSEC, in the liver.


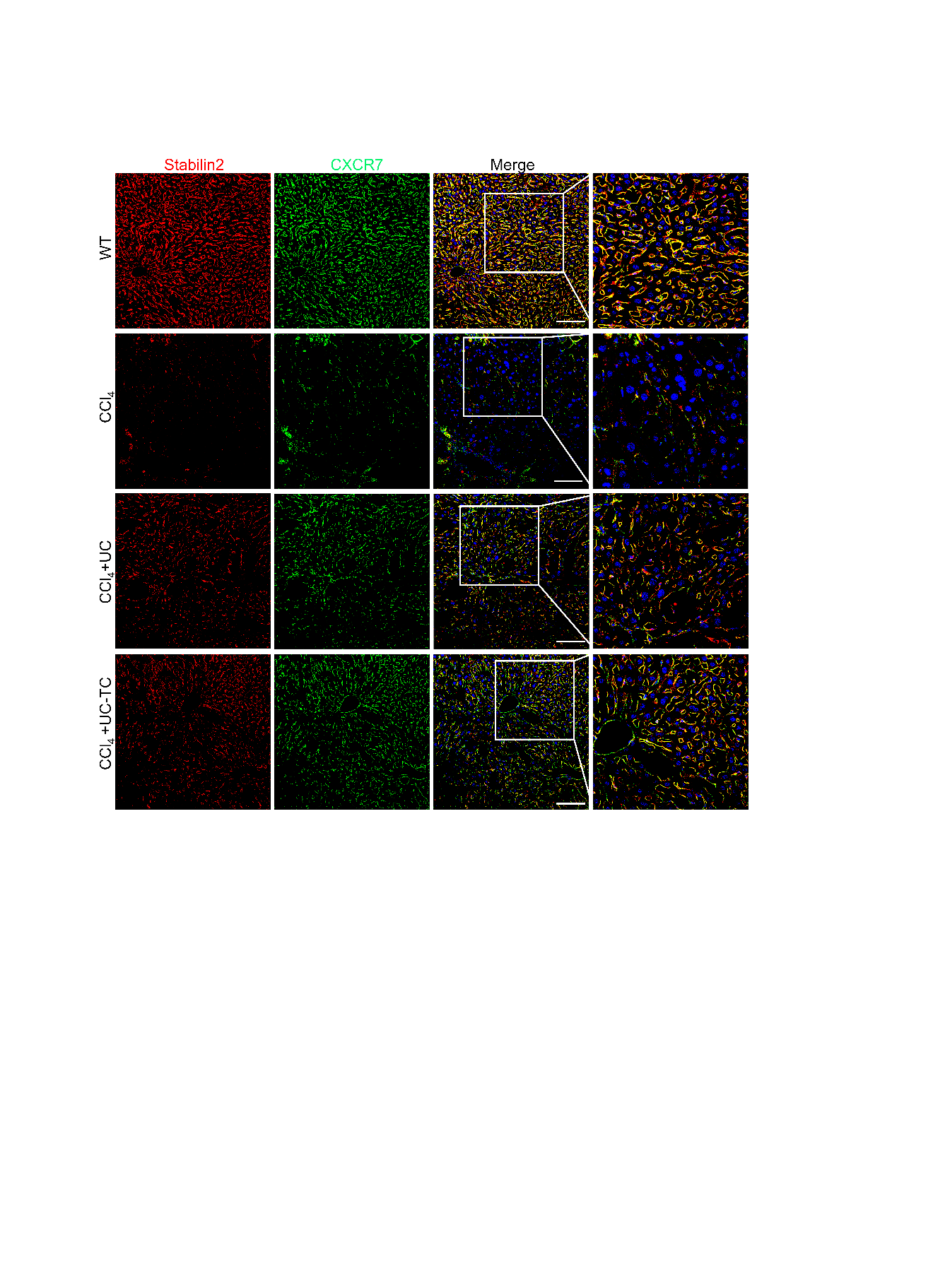


**sFigure 7** CXCR7 co-stained with Stabilin2 in liver

CXCR7 (green) was co-stained with Stabilin2 (red) in the liver, images were captured using a laser scanning confocal microscope (Olympus FluoViem FV 1000, Tokyo, JPN). The results showed that CXCR7-positive signals mainly colocalized with Stabilin2-positive of LSEC, especially in WT group. Scale bar: 100 μm.


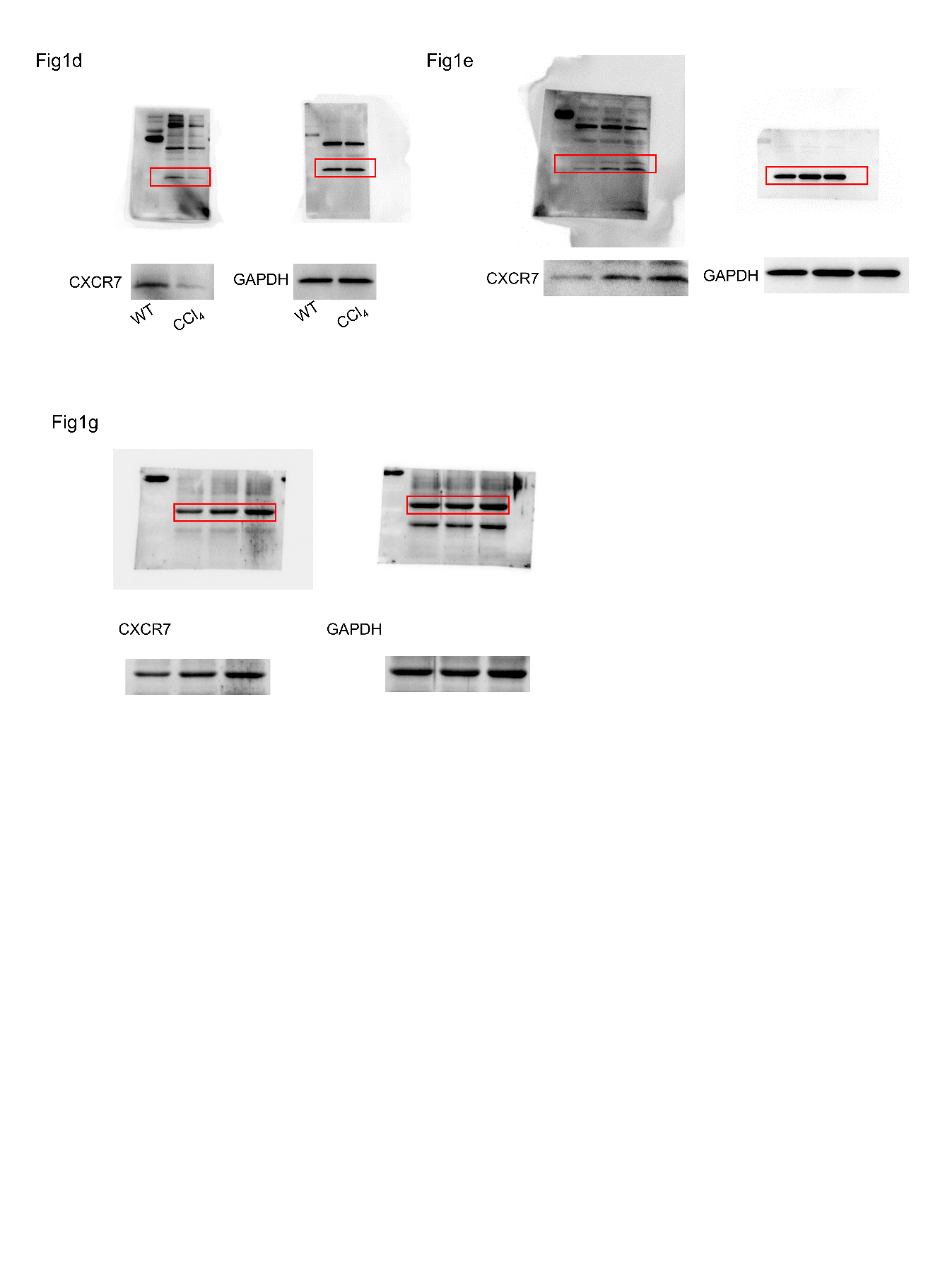


**sFigure 8** Images of the original blots shown in the Fig. 1. Boxes indicate cropped regions.

**Supplementary Table 1**

**Routine blood examinations of UC-MSCs implantation**

Blood was collected in an EDTA anticoagulation tube immediately at the time of sacrifice. Then, routine blood examinations were analyzed for complete blood counts (CBCs), including white blood cell (WBC), red blood cell (RBC) counts, haemoglobin, and platelet counts.

|  | WT | CCl_4_ | CCl_4_+UC-MSCs | CCl_4_+UC-MSC-TC | Unit |
| --- | --- | --- | --- | --- | --- |
| WBC | 7.60±0.89 | 6.73±2.77 | 6.07±070 | 5.90±1.00 | 10^9/L |
| Lymph# | 6.90±1.20 | 5.55±2.05 | 5.85±2.71 | 4.38±0.87 | 10^9/L |
| Mon# | 0.20±0.00 | 0.33±0.22 | 0.38±0.35 | 0.20±0.00 | 10^9/L |
| Gran# | 1.50±0.10 | 2.58±1.98 | 2.53±2.39 | 1.33±0.26 | 10^9/L |
| Lymph% | 80.00±1.90 | 68.38±10.65 | 70.55±9.10 | 73.40±4.63 | % |
| Mon% | 2.60±0.30 | 3.70±1.22 | 3.88±1.10 | 3.53±0.69 | % |
| Gran% | 17.40±1.60 | 27.93±9.44 | 25.58±8.05 | 23.08±4.00 | % |
| RBC | 9.21±0.08 | 6.28±0.55 | 7.63±0.49 | 8.33±0.36 | 10^12/L |
| HGB | 132.50±0.50 | 82.25±11.53 | 104.25±10.44 | 117.00±6.16 | g/L |

**Materials and methods**

**Identification the characteristics of UC-MSCs and LSEC**

For immunophenotypic characterization of UC-MSCs, UC-MSCs (passage 5) were incubated with antibodies: CD3-FITC (eBioscience, 11-0319-42), CD45-FITC (eBioscience,11-9459-42), CD73-PE (eBioscience, 12-0739-42), CD90-PE, and CD105-PE (sFig. 1**a**), and were examined by flow cytometry (Beckman CytoFLEX S, USA). For clone formation units, UC-MSCs were seeded with 3000 cells in 6-well plates, and replaced with fresh culture medium every 3 days. After 10 days of culture, UC-MSCs were fixed with 4% paraformaldehyde and stained with 1% crystal violet at room temperature for 10 minutes. The clone formation units were observed under a microscope (sFig. 1**b**). For osteogenic differentiation, UC-MSCs were seeded with 1×10^5^ cells in 12-well plates. When the cell fusion reached more than 95%, UC-MSCs were induced with osteogenic medium and changed 2-3 times per week for 21 days. The UC-MSCs were then fixed with 4% paraformaldehyde and stained with 1% Alizarin red at room temperature for 3 minutes. The mineralization nodules were observed under a microscope (sFig. 1**c**).

To identify LSEC-specific fenestra structures on cell surface, LSEC were harvested and fixed in 4% glutaraldehyde and 0.1 M PBS (pH 7.4) for 24 hours, and then dehydrated in a gradient of 35%, 50%, 70%, 80%, 90%, and 100% ethanol. Samples were dried at room temperature, coated with gold, and captured images using a SEM (S-4800, Hitachi) at a voltage of 5.0 kV (sFig. 1**d**).

**UC-MSCs co-culture with T lymphocytes**

T lymphocytes were isolated from mice spleens. Spleens were placed on 100-mesh strainer, and crushed with a syringe plunger. The grinded cells were rinsed with PBS, centrifuged to pellet cells, and then incubated with red blood cell lysis buffer at room temperature for 10 minutes. The cells were resuspended in RPMI 1640 containing with 2 μg/mL CD28 antibody and cultured in 9 cm dishes with precoated with 5 μg/mL CD3 antibody at 37 °C with 5% CO_2_ for 2-3 days. Meanwhile, UC-MSCs were seeded in 6-well plates, and 2×10^5^ UC-MSCs were treated with or without 30 μM TC14012 for 1 day before co-culture with T lymphocytes. Then, 1×10^6^ activated T lymphocytes were transferred onto UC-MSCs and directly co-cultured for 48 hours. The T lymphocytes were then harvested, and cell cycle or cell apoptosis were examined by flow cytometry (Beckman CytoFLEX S, USA). For cell cycle or cell apoptosis assays, the cells were performed with a Cell Cycle and Apoptosis Analysis Kit (Yeasen, 40301ES60, CHN) or an Annexin V-FITC/PI Apoptosis Detection Kit (Yeasen Biotechnology, 40302ES50, CHN) according to the manufacturer’s instructions.

**Primary LSEC isolation**

Primary LSEC were isolated from C57BL/6 mice that were injected with CCl_4_. Briefly, LSEC were initially isolated from the liver by perfusion with collagenase (LiberaseTM, Sigma). Hepatocytes (parenchymal cells) were removed by centrifugation at 62 g at 4 °C at low speed. To isolate LSEC, a gradient density sedimentation method was used with non-parenchymal resting cells on 25–50% Percoll, and the cells were purified with LSEC-specific CD146-based isolation on magnetic MicroBeads (MACS, MiltenyiBiotec, Germany). The isolated LSEC were cultured in DMEM with 10% FBS (Sijiqing, CHN), 2 mM L-glutamine (Gibco, USA), 100 U/mL penicillin/streptomycin (Beyotime, CHN), and incubated at 37 °C with 5% CO_2_.

**AST and ALT tests**

Blood was collected immediately at the time of sacrifice and the serum was separated by centrifugation at 5000 g at 4 °C for 10 minutes. Aspartate aminotransferase (AST) and alanine aminotransferase (ALT) were assessed by using AST Assay Kit or ALT Assay Kit from NJJCBIO Company (NJJCBIO, CHN), respectively, according to the manufacturer's instructions.

**Biosecurity test**

To assess the safety of UC-MSCs transplantation *in vivo*, mice from fibrotic groups were sacrificed at the endpoints. Blood and major organs, including the hearts, lungs, kidneys, and spleens were harvested. Routine blood examinations were analyzed for complete blood counts (CBCs), including white blood cell (WBC) and red blood cell (RBC) counts, haemoglobin, and platelet counts. Additionally, blood serum was collected, and lactic dehydrogenase (LDH), creatine kinase (CK), blood urea nitrogen (BUN), and creatinine (Cr) were examined by performed using enzyme-linked immunosorbent assay (ELISA) kits. Histological analysis of tissues was performed using hematoxylin and eosin (H&E) staining.

**Histological analysis**

After sacrificing mice, the livers, hearts, lungs, kidneys, and spleens were fixed in 4% formaldehyde, embedded in paraffin, and sectioned (6 μm). Histopathological analysis of these sections was performed by Leica autostainer XL using H&E. To determine collagen deposition in liver, Masson trichrome staining was performed with a kit from Solarbio (Solarbio, Peking, China) as instructed by the manufacturer. The images were captured using an Olympus microscope and analyzed using the Image J software.
